# Comparative Phytochemical Profiling of Essential Oils from Selected Abies Species and Analysis of Their Antifungal and Antiradical Activity

**DOI:** 10.3390/pharmaceutics18010026

**Published:** 2025-12-25

**Authors:** Rizvangul Ayupova, Emil Svajdlenka, Milan Zemlicka, Galiya Ibadullayeva, Karlygash Raganina, Urziya Alimova, Shamshabanu Nokerbek, Rauan Botabayeva, Lashyn Kiyekbayeva, Serzhan Mombekov

**Affiliations:** 1 Department of Pharmaceutical Technology, S.D. Asfendiyarov Kazakh National Medical University, Tole-bi 94, Almaty 050012, Kazakhstanraganina.k@kaznmu.kz (K.R.); alimova.u@kaznmu.kz (U.A.); 2 Department of Natural Drugs, Faculty of Pharmacy, University of Veterinary and Pharmaceutical Sciences, 61200 Brno, Czech Republic; 3 School of Pharmacy, S.D. Asfendiyarov Kazakh National Medical University, Tole-bi 94, Almaty 050012, Kazakhstan; 4 Medical College, South Kazakhstan Academy of Medicine, Shymkent 160019, Kazakhstan; 5 Department of Engineering Disciplines of Good Practices, School of Pharmacy, Kazakh National Medical University, 88 Tole Bi Street, Almaty 050012, Kazakhstan

**Keywords:** *Abies*, essential oils, antifungal activity, antiradical activity, bornyl acetate, GC–MS, phytochemical profiling

## Abstract

**Background/Objectives:** The essential oils of *Abies* species possess a complex chemical composition and pronounced biological activity. However, comparative studies of interspecies differences and on the influence of extraction methods on their chemical profile and pharmacological properties are limited. Such data are necessary for identifying the most promising species and optimizing essential oil production technologies for pharmaceutical applications. The aim of this study was to comparatively evaluate the essential oils of seven *Abies* species obtained by steam distillation and to analyze the effects of three extraction methods on the chemical and biological characteristics of *Abies sibirica* L. oil. **Methods:** The essential oils of seven *Abies* species were isolated by steam distillation. For *A. sibirica*, supercritical CO_2_ extraction and microwave-assisted steam distillation (MASD) were additionally used. Chemical composition was determined by GC-MS. Statistical analysis included ANOVA, PCA and hierarchical cluster modeling, and non-parametric tests. Antifungal activity was assessed against *Candida albicans*, and antiradical activity was assessed using densitometric analysis. **Results:** GC-MS analysis revealed significant differences in chemical composition between species and extraction methods. The main compounds were bornyl acetate, α-pinene, and camphene. ANOVA revealed significant differences in bornyl acetate and β-pinene content between species (*p* < 0.001) and methods (*p* < 0.01). PCA and clustering identified a bornyl acetate-rich chemotype (*A. sibirica*, *A. grandis*, *A. lowiana*). *A. sibirica* oil, obtained by MASD, exhibited high antifungal activity (82% inhibition), similar to that of 5-flucytosine (95%). Antiradical activity correlated with monoterpenes corresponding to peaks 2 and 7 of the densitogram. **Conclusions:** This study demonstrates that the species and extraction method significantly determine the chemical profile and biological properties of *Abies* oils. *A. sibirica* oil, obtained by MASD, demonstrated the highest activity, highlighting its potential as a source of biologically active compounds.

## 1. Introduction

Essential oils from *Abies* species (fir trees) have long been valued for their uses in aromatherapy, traditional medicine, cosmetics, and industry, due to their distinctive chemical profiles and broad bioactivities [1,2,3,4,5,6,7]. Traditionally, they were appreciated for their resinous fragrance, stress-relieving and concentration-enhancing effects, and ethnobotanical applications as antiseptic, anti-inflammatory, expectorant, and analgesic agents, including treatment of respiratory disorders, musculoskeletal pain, and wound healing [5,6,7].

Modern studies sopport these traditional uses, showing that *Abies* essential oils possess strong antimicrobial activity, particularly against Gram-positive bacteria, along with antifungal, anti-inflammatory, and antioxidant properties [2,3,6,7,8,9]. These effects are mainly attributed to monoterpenes (α-pinene, β-pinene, camphene, limonene) and oxygenated derivatives (bornyl acetate, borneol), which modulate oxidative stress, inhibit microbial growth, and exert immunomodulatory effects [2,3,7]. Emerging evidence also suggests broader pharmacological activities, including anticancer, antihypertensive, antitussive, antifungal, and CNS-modulating effects [8,9,10,11,12,13].

In cosmetics, fir oils are used for skin and hair care, improving microcirculation, reducing inflammation, and providing antioxidant protection [4,6,14]. Industrially, they serve as natural, sustainable alternatives to synthetic additives in perfumery, cleaning products, and food systems [3,4].

The genus *Abies* includes over fifty species across Asia, Europe, North and Central America, and North Africa, with substantial interspecific and geographical variation in essential-oil composition [2,4,6,15]. For instance, *A. cephalonica* (Greece) is rich in β-pinene and camphene [7]; *A. balsamea* (North America) contains high α-pinene and bornyl acetate [4,6]; *A. koreana* (East Asia) is dominated by δ-3-carene [2,16,17,18]. These chemotypic differences result from genetic and environmental factors, such as altitude, climate, and soil, affecting bioactivity and industrial potential [5].

Despite this diversity, data on several species remain limited, particularly *A. sibirica* from East Kazakhstan, a unique biogeographical region. Recognising its medicinal potential, a novel gel formulation based on *A. sibirica* was investigated [15]. Comparative analyses across multiple species under controlled conditions, integrating chemical profiling, chemometrics, and biological evaluation, remain scarce [7,15]. The effects of ontogeny and extraction methods on essential-oil composition also require further study [19,20].

The present study provides the first integrated chemical and biological assessment of *A. sibirica* essential oil from East Kazakhstan, alongside six other Central European *Abies* species. Using GC–MS, multivariate chemometrics, and antifungal testing, we aimed to: (i) characterise species-specific chemical profiles; (ii) assess ontogenetic variations; (iii) evaluate the impact of extraction techniques; and (iv) investigate antifungal and antiradical activity.

By clarifying chemotaxonomic relationships, bioactive potential, and processing considerations, this work advances our understanding of *Abies* essential oils and supports their sustainable use in pharmaceutical, cosmetic, and industrial applications.

## 2. Materials and Methods

### 2.1. Plant Material and Authentication

Branch tips (30–40 cm) of *Abies sibirica Ledeb*. were collected in June 2024 from the East Kazakhstan region, Republic of Kazakhstan (Altai, Tarbagatai, and Dzungarian Alatau mountain ranges; GPS coordinates: 49°34′44″ N, 82°36′20″ E; altitude: 1289 m a.s.l.). The plant material was taxonomically identified by Dr Elmira Sailauhanovna Sametova, Senior Researcher at the Institute of Botany and Phytointroduction, Almaty. A voucher specimen (accession no. ALA-2024-006) was deposited in the herbarium of the Institute of Botany and Phytointroduction, Almaty.

Samples of six additional *Abies* species (*A. pinsapo*, *A. grandis*, *A. concolor*, *A. nordmanniana*, *A. lowiana*, and *A. alba*) were collected in July 2024 from the Central Botanical Garden, Brno, Czech Republic. These were authenticated by staff of the Central Botanical Garden, and corresponding voucher specimens were deposited under accession numbers BRNO-12345 to BRNO-12350.

All collections were conducted in compliance with national regulations. Fresh needles were air-dried at 20–22 °C in a well-ventilated room for seven days prior to extraction. In this study, the terms ‘1-, 3-, and 5-year-old tissues’ refer to the age of branch segments, determined by annual growth rings, rather than the chronological age of the trees themselves.

### 2.2. Essential Oil Extraction

Essential oils of six species of Abies were obtained by steam distillation (3 h, 250 g of fresh needles) according to the European Pharmacopoeia [20], with three replicates for each species and age group. The oil yield (% *v*/*w*) was calculated as the ratio of the oil volume to the mass of the plant material, multiplied by 100. The oils were dried over anhydrous sodium sulphate, filtered and stored at 4 °C until analysis.

The essential oil yield (% *v*/*w*) was calculated using the following formula:

$$Yield\left(\%\frac{v}{w}\right)=\frac{Volume\;of\;essential\;oil\;(mL)}{Weight\;of\;fresh\;plant\;material\;(g)}\times 100$$

*Abies sibirica* essential oils were extracted using three methods: water steam distillation, supercritical CO_2_ extraction (40 °C, 10 MPa) and microwave steam distillation (400 W, 30 min). The chemical composition was analysed using GC–MS.

### 2.3. Gas Chromatography–Mass Spectrometry (GC–MS) Analysis

GC–MS analyses were performed on an Agilent 7890A with a 5975C VL detector and a 7683B autosampler (Agilent Technologies, Santa Clara, CA, USA). Separation was performed on a Varian CP9103 column (60 m × 0.25 mm, 1.4 μm). Temperature programme: 40 °C (4 min) → 270 °C at a rate of 4 °C/min (2.5 min), total duration—64 min. Injector and transfer line temperatures were 260 °C and 280 °C, respectively; carrier gas was He, 1.2 mL/min. The mass spectrometer operated in EI mode (70 eV), range 29–250 *m*/*z*, solvent delay 5 min. Injections 1 μL, split 1:100.

### 2.4. Compound Identification

Retention indices (RI) were determined using a homologous series of n-alkanes (C7–C35; Sigma-Aldrich, Steinheim, Germany) under the same chromatographic conditions. Compounds were identified by comparing their mass spectra with the NIST05 and WILEY138 libraries, as well as by comparing the calculated RIs with published data [21]. Only compounds with a match factor ≥ 85% were accepted as reliably identified. The relative percentage of each component was calculated based on peak areas without correction factors. Each essential oil sample was analysed in triplicate, with RSDs of the main components < 5% [22].

### 2.5. Antifungal Activity Assay

The antifungal activity of *Abies sibirica* essential oils was studied on *Candida albicans* strains under aseptic conditions. The oils were dissolved in dimethyl sulfoxide and 0.9% NaCl and added to 96-well microplates (100 μL per well). The maximum test concentration was 256 μg/mL; 5-flucytosine (1 μg/mL) was used as a positive control. Fungal growth was assessed by optical density at 600 nm for 48 h, and relative growth was calculated by the area under the curve relative to the control [15].

### 2.6. Antiradical Activity

#### HPLC with Post-Column Derivatisation

**Figure 1:**
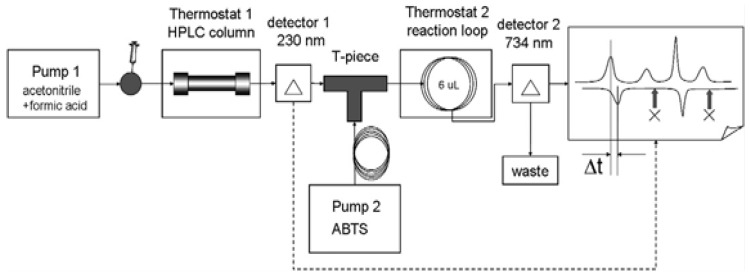
Determination of antiradical activity of the essential oil from *Abies sibirica* L. using HPLC.

The antiradical activity of *Abies sibirica* essential oil was determined by HPLC with post-column derivatisation. This configuration ensured a stable flow of the reaction mixture and increased the sensitivity and selectivity of the analysis [23] (Figure 1).

### 2.7. Statistical Analysis

All experiments were performed in triplicate, and data are presented as mean ± SD. Statistical analysis was performed using one-way ANOVA with Tukey’s test (*p* ≤ 0.05) in GraphPad Prism v.10.0. Due to the small sample size (*n* = 3), ANOVA was used for preliminary comparative analysis; larger studies will allow the use of advanced statistical models.

Given the small sample size for essential oil from *Abies sibirica* L. obtained by three methods (WSD, SC-CO2, MASD), non-parametric tests were used.

## 3. Results and Discussion

### 3.1. Chemical Composition of Abies Essential Oils (Steam Distillation)

Steam-distilled essential oils from seven *Abies* species were pale yellow in colour, exhibiting a characteristic balsamic-pine aroma. GC–MS analysis identified more than 90% of the total volatile constituents (see Supplementary File S1, Figure S1, Table S1), which were dominated by monoterpene hydrocarbons (*α-pinene*, *camphene*, *β-pinene*, *limonene*) and oxygenated monoterpenes (bornyl acetate, borneol). This terpene-rich composition is consistent with previous reports on *Abies* species from Europe, Asia, and North America, where α-pinene and camphene typically constitute the principal volatile fraction [2,7,15,17,22,23,24].

Species-specific chemical markers were identified: α-phellandrene in *A. nordmanniana*, α-campholenal in *A. lowiana*, cadinol in *A. pinsapo*, absence of β-myrcene in *A. pinsapo*, and absence of β-phellandrene in *A. alba*. Such characteristics have been recognised as powerful tools for chemotaxonomic differentiation and authenticity testing in conifers. Comparable marker-based approaches have been successfully employed to discriminate Mediterranean firs such as *A. cephalonica* [7] and Balkan *Abies* populations [4]. Moreover, molecular markers have confirmed distinct species boundaries among *A. nordmanniana*, *A. alba*, and *A. pinsapo* [25,26].

These findings underscore the value of integrating chemical and genetic markers for reliable species authentication and traceability in essential oil research. Representative GC–MS chromatograms of essential oils from the following *Abies* species were produced: (a) *A. alba*; (b) *A. concolor* (1 year); (c) *A. concolor* (3 years); (d) *A. grandis* (1 year); (e) *A. grandis* (3 years); (f) *A. lowiana* (1 year); (g) *A. lowiana* (3 years); (h) *A. lowiana* (5 years); (i) *A. nordmanniana* (1 year); (j) *A. nordmanniana* (3 years); (k) *A. pinsapo* (1 year); (l) *A. pinsapo* (3 years); (m) *A. sibirica*. All chromatograms are provided as part of this article Figure S1. (see Supplementary File S1).

Comparative analysis highlights the distinct chemotypes of the Eurasian *Abies* species examined. *A. cephalonica* oil from Mt. Ainos, Greece, contains 63.9% monoterpene hydrocarbons (β-pinene 26.9%, α-pinene 10.1%, camphene 9.2%) and 12.5% bornyl acetate [7], whereas *A. sibirica* exhibits a markedly higher bornyl acetate content (~31%), and *A. concolor* displays exceptionally elevated β-pinene levels (~49%). Such variations emphasise the combined influence of geography, genetic background, and environmental factors on *Abies* secondary metabolism, with important implications for both their ecological adaptation and industrial utilisation [4,25,26].

### 3.2. Effect of Vegetative Age (Ontogenetic Variation)

**Figure 2:**
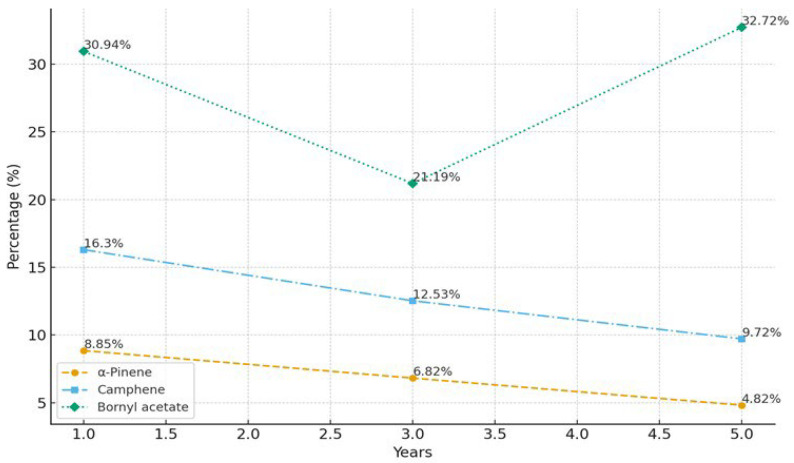
Changes in α-pinene, camphene, and bornyl acetate content in *A. lowiana* essential oil at 1, 3, and 5 years of age.

Focusing on branch age rather than the chronological age of the whole tree enables assessment of developmental changes in metabolite allocation within actively growing tissues, which is particularly relevant to essential oil biosynthesis. Ontogenetic variation in essential oil composition was evident across the sampled age classes (1-, 3-, and 5-year-old tissues; Figure 2). Monoterpene hydrocarbons predominated in younger material, whereas mature tissues showed a marked enrichment in oxygenated monoterpenes. In *A. lowiana*, for instance, *α*-pinene decreased from 8.85% at 1 year to 4.82% at 5 years (*p* < 0.05), while bornyl acetate increased to 32.72%, reflecting a metabolic shift from highly volatile to more stable oxygenated terpenoids. More detailed information is provided in Supplementary File S1, Table S1.

This ontogenetic trend mirrors patterns reported in other conifers, such as *Pinus sylvestris* and *Picea abies*, where juvenile foliage produces high levels of α- and *β*-pinene as rapidly synthesised volatile defences against herbivores and pathogens [27,28,29]. With maturation, metabolic investment shifts towards oxygenated monoterpenes such as bornyl acetate—compounds associated with long-term defence, antimicrobial protection, and oxidative stability [30]. Such age-related metabolic reprogramming likely reflects an adaptive strategy in secondary metabolism, prioritising transient volatile deterrents during early establishment and progressively transitioning to more durable chemical barriers as structural development proceeds [29].

From both functional and industrial perspectives, this developmental trajectory holds considerable significance: juvenile oils, rich in bright, volatile monoterpenes, are particularly suited to perfumery and aromatherapy, whereas oils from mature trees, enriched in oxygenated monoterpenes, exhibit superior stability and bioactivity for pharmaceutical and preservative applications [7].

These findings emphasise the importance of age-targeted harvesting to optimise essential oil composition and functional value.

### 3.3. Influence of Extraction Method (A. sibirica)

The extraction technique exerted a marked influence on the composition of *A. sibirica* essential oil (Table 1). Water-steam distillation (WSD) produced oils dominated by volatile monoterpenes, including *α*-pinene (15.81%), camphene (15.83%), and 3-carene (15.04%). This composition is consistent with the hydrothermal co-distillation process, which preferentially extracts highly volatile constituents while promoting partial hydrolysis or loss of thermolabile compounds [19,30]. A more detailed version of the essential oil component composition can be found in Supplementary File S2.

In contrast, according to Table S2 in Supplementary File S2, extraction using supercritical CO_2_ (SC–CO_2_) produced a chemically distinct profile, enriched in bornyl acetate (40.0%) and sesquiterpenes such as copaene (0.063%). This reflects the superior solvating capacity and tunable density of supercritical CO_2_, which efficiently recovers heavier and oxygenated molecules while preserving their structural integrity [31,32,33,34,35,36,37,38,39,40,41]. Such properties render SC–CO_2_ particularly suitable for obtaining bioactive-rich extracts intended for pharmaceutical and preservative applications, as demonstrated in other Pinaceae species [42,43,44,45].

Microwave-assisted steam distillation (MASD) yielded intermediate profiles, characterised by elevated santene (5.47%) and oxygenated compounds such as camphor (0.71%), suggesting enhanced cell wall disruption and efficient release of semi-volatile constituents under rapid volumetric heating [36,43,44].

GC-MS chromatogram of volatile components of essential oils extracted from *Abies sibirica* L. using the methods of water-steam distillation (A), carbon-dioxide extraction (B), and microwave-assisted steam distillation (C) (Figure S2, Supplementary File S2).

From an industrial standpoint, the ability to tailor the composition of *A. sibirica* oil through the choice of extraction method holds considerable strategic significance. SC–CO_2_ extracts, rich in bornyl acetate and sesquiterpenoids, are particularly well-suited for pharmaceutical and therapeutic formulations, whereas water-steam distilled (WSD) oils, characterised by high *α*-pinene content, cater effectively to perfumery and aromatherapy markets.

Moreover, the solvent-free and environmentally benign nature of SC–CO_2_ extraction aligns with the growing emphasis on sustainable essential oil production [38]. A limitation of the present study is that alternative extraction techniques (SC–CO_2_, MASD) were applied solely to *A. sibirica*. While this demonstrates the potential of advanced extraction methods for this species, future research should investigate whether comparable compositional shifts occur across other *Abies* taxa to enable broader generalisation of these findings.

### 3.4. Antifungal Activity of Essential Oil from Abies sibirica L.

There are several reliable studies confirming that the essential oils of certain Abies species (primarily *A. holophylla* and *A. alba*) exhibit notable antifungal activity. However, the level of activity depends on the species, the extraction method and the conditions of application, which makes it difficult to draw universal conclusions [46,47,48,49,50,51,52,53].

The experiment was conducted under aseptic conditions. To evaluate the antifungal activity of essential oils from *Abies sibirica* L., reference strains of *Candida albicans* were obtained from the Laboratory of the Department of Infectious Diseases and Microbiology, Veterinary and Pharmaceutical University, Brno, Czech Republic. Samples of essential oils were dissolved in dimethyl sulfoxide and 0.9% saline solution and subsequently introduced into flat-bottomed 96-well microplates.

**Figure 3:**
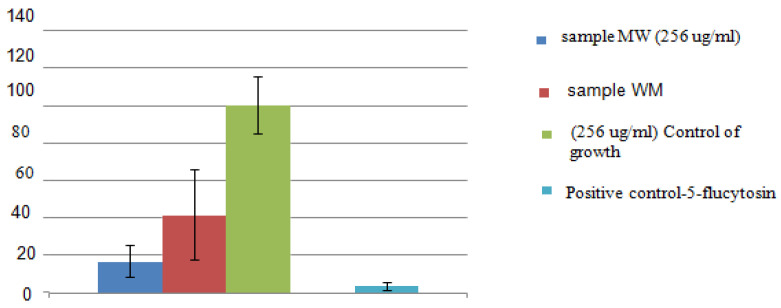
Antifungal activity of essential oils of *Abies sibirica* L.

The fungal inoculum was adjusted using a multichannel pipette to achieve a final volume of 100 µL per well. The highest fungistatic concentration of the oil extract was 256 µg/mL. 5-Flucytosine (1 µg/mL) served as a positive control. Fungal growth was monitored by measuring optical density at 600 nm using a microplate reader (BMG Labtech Reader, Ortenberg, Germany) at 37 °C over a 48-h period. Quantitative comparison of the areas under the growth curves across 48 h provided relative growth values, expressed as a percentage of the untreated control (Figure 3).

**Figure 4:**
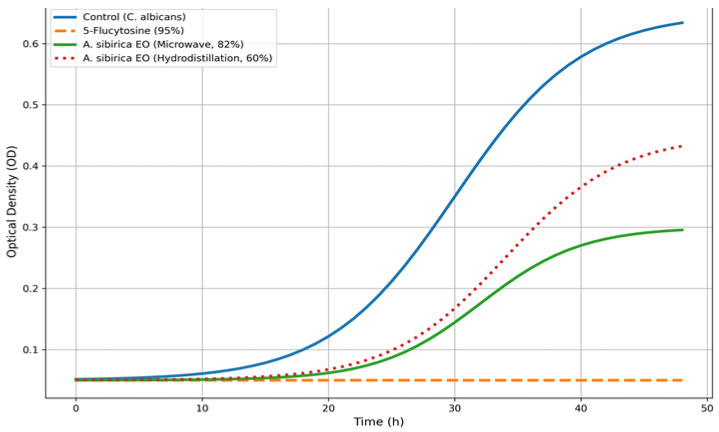
Growth curves of *Candida albicans*.

Antifungal activity was further assessed using a SPECTROstar Omega (BMG LABTECH, Ortenberg, Germany) microplate reader. The results of the antifungal activity assays for essential oils from *Abies sibirica* L. are presented in Figure 3 and Figure 4.

As shown in Figure 3 and Figure 4, the synthetic antifungal drug 5-flucytosine was used as a reference standard. The antifungal activity of 5-flucytosine against *Candida albicans* was 95%, whereas the essential oil of *Abies sibirica* L. obtained by the microwave extraction method exhibited an activity of 82%. This represents a notably high value for a biologically active compound of plant origin, particularly when compared with a synthetic antifungal agent. The essential oil of *A. sibirica* L. obtained via hydrodistillation demonstrated comparatively lower activity, achieving 60% inhibition against *C. albicans.*

Overall, the antifungal assay revealed that the essential oil of *A. sibirica* L. produced by microwave extraction possesses substantial antifungal efficacy against *Candida albicans*. The minimum inhibitory concentration (MIC) for the *A. sibirica* essential oil was determined to be 300 µg/mL.

### 3.5. Antiradical Activity of Essential Oil from Abies sibirica L.

Essential oils of *Abies* exhibit moderate to pronounced radical-scavenging activity in standard assays (DPPH, ABTS, FRAP, etc.), although the results vary considerably between species, plant parts, seasons, and extraction methods.

The monoterpenes (α-pinene, limonene, camphene, bornyl acetate, and others) play the leading role in this activity, while oxygen-containing terpenoids contribute to a lesser extent. Certain oils with a high content of limonene/α-pinene have shown comparatively strong activity in ABTS and DPPH tests. However, the relationship between composition and activity is not always linear: a quantitative predominance of a single component may result in lower activity than that of a mixture, owing to synergy or antagonism between constituents [54,55].

Some authors have developed an effective method for the simultaneous identification and detection of antioxidant components in Chrysanthemum flowers [55,56].

**Figure 5:**
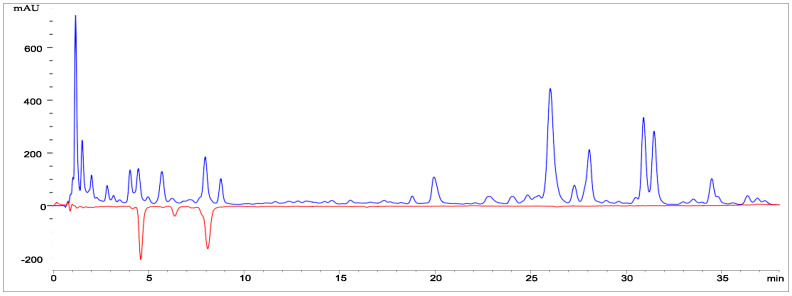
Chromatograms of the essential oil of *Abies sibirica* L. under experimental conditions: column II, sample volume was 10 μL, flow rate Q1 = 1 mL/min, Q2 = 0.53 mL/min, column temperature (thermostat 1) 300 °C, temperature of thermostat 2 was 500 °C. The dotted trace (detector 2, negative signal) represents radical scavenger signals. Time difference 1.5 s.

Natural antioxidants play a crucial role in regulating free radical processes within the body, exerting a significant influence on overall physiological balance and contributing to the strengthening of the human immune system. Essential oils derived from wild-growing coniferous species are also known to exhibit antioxidant and antiradical activities; however, their antiradical activity (ARA) has been relatively little studied to date. The chromatogram of the essential oil is presented in Figure 5.

**Figure 6:**
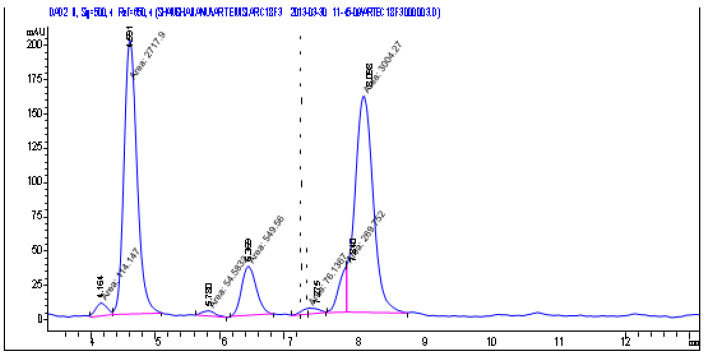
Densitograms of the chromatograms of essential oil from *Abies sibirica* L.

The chromatogram shows peaks corresponding to the main components of the analyzed essential oil (Figure 6):

1. The first major peak appears in the region of approximately 5.2–5.4 min. Its high intensity (about 180–200 mAU) indicates that this component is one of the dominant constituents of the oil.
2. The second significant peak is located at around 8.0–8.3 min. Its height is comparable to that of the first major peak, which likewise suggests a high concentration of the corresponding compound.
3. Between the large peaks, several smaller peaks of lower intensity are visible in the range of 6–7 min. These represent minor components present in smaller amounts.
4. The baseline is stable, without significant fluctuations, confirming proper chromatographic separation and the absence of interference.

Thus, the chromatogram demonstrates clear separation of the major and minor components of the essential oil, with two prominent peaks indicating the predominant substances in its composition.

The densitogram displays two types of signals: a main positive signal (detector 1) and a negative signal corresponding to radical-scavenging activity (detector 2). The primary curve (blue line) represents the distribution of individual components of the essential oil as a function of retention time. Several distinct peaks are evident on the graph, corresponding to the major terpene constituents characteristic of *Abies sibirica* essential oil. The highest signal intensity is observed within the range of 8–25 min, indicating the presence of volatile mono- and sesquiterpenes with varying polarity.

The dotted red line (negative values) represents the activity of compounds capable of scavenging free radicals. The presence of negative peaks within the 10–20 min region indicates that certain constituents of the oil exhibit pronounced radical-scavenging activity.

Thus, the densitogram not only illustrates the chemical profile of *Abies sibirica* L. essential oil but also confirms the presence of biologically active compounds capable of neutralising free radicals. These findings highlight the potential antioxidant properties of the oil and its prospective applications in pharmaceutical and cosmetic formulations.

**Figure 7:**
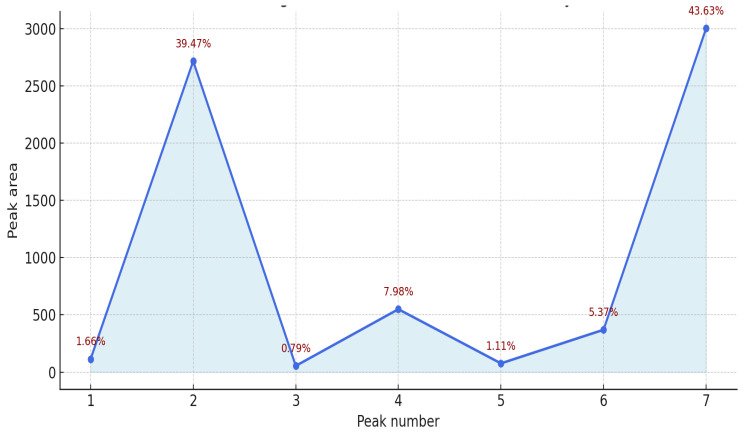
Densitogram with indicated antiradical activity of essential oil from *Abies sibirica* L.

The results presented in Table 2 and Figure 7 illustrate the antiradical activity profile of the essential oil obtained from *Abies sibirica* L. The total antioxidant capacity, expressed in Trolox equivalents, was 1609.0 µL/L, corresponding to 100% relative activity.

Among the identified components, peaks 2 and 7 exhibited the highest contributions to the overall antiradical activity, accounting for 39.47% and 43.63%, respectively. Together, these two fractions represented more than 83% of the total activity, indicating that a limited number of major constituents predominantly determine the oil’s antioxidant potential.

Moderate activity was recorded for peaks 4 and 6, contributing 7.98% and 5.37%, respectively, while peaks 1, 3, and 5 made only minor contributions (each below 2%).

Overall, these findings suggest that the antioxidant potential of *Abies sibirica* essential oil is largely governed by a few dominant terpene constituents, likely characterised by strong electron-donating and radical-scavenging properties. Further chemical identification of these peaks is recommended to elucidate the specific compounds responsible for the observed activity.

### 3.6. Statistical Analysis

ANOVA and PCA methods were used to process *Abies* species data.

This trend reflects the well-documented variability of early-branch pathway monoterpenes, the biosynthesis of which is strongly influenced by species-specific polymorphisms in terpene synthase genes [31,32,33]. In contrast, β-pinene, bornyl acetate, and β-caryophyllene exhibited no significant interspecific variation (*p* > 0.05), consistent with their roles as downstream, metabolically stable end-products of the terpenoid biosynthetic pathway [19].

**Figure 8:**
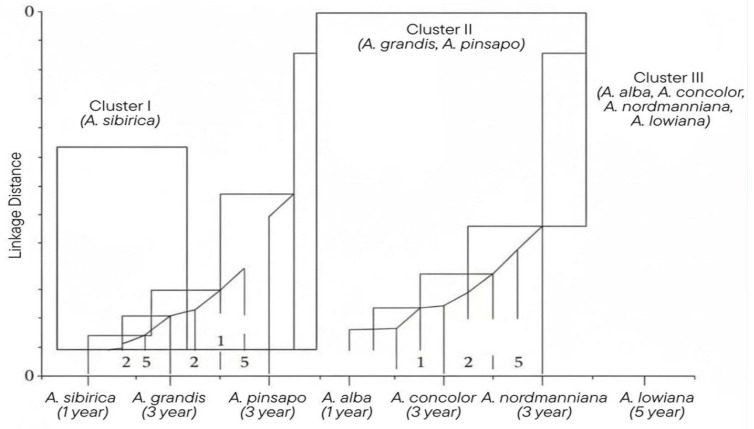
Hierarchical clustering (Ward linkage) of essential oil composition across seven *Abies* species at different ages. Three main clusters are evident: Cluster I (*A. sibirica*), Cluster II (*A. grandis*, *A. pinsapo*), and Cluster III (*A. alba*, *A. concolor*, *A. nordmanniana*, *A. lowiana*).

Principal component analysis (PCA) and hierarchical clustering (Table 3, Figure 8) delineated three distinct chemotypic clusters: (i) *A. sibirica*, forming a unique group characterised by elevated camphene and bornyl acetate levels; (ii) *A. pinsapo* and *A. grandis*, exhibiting substantial compositional similarity; and (iii) *A. alba*, *A. concolor*, *A. nordmanniana*, and *A. lowiana*, forming a broader assemblage (see Supplementary File S1, Table S1). This clustering pattern accords with previous chemometric investigations of essential oils and conifer terpenes [34], and corresponds closely to established chemotaxonomic frameworks for Mediterranean and Eurasian firs [7,15]. Furthermore, integration with molecular datasets has confirmed that these chemical groupings frequently reflect evolutionary lineages and geographical adaptations within the genus *Abies* [25,35].

The distinct positioning of *A. sibirica* highlights its unique chemotype within the genus, conferring both scientific and applied significance. Such chemometric differentiation serves as a powerful tool for species authentication and traceability, addressing issues of adulteration and mislabelling within the essential oil industry. Moreover, the identification of camphene and α-pinene as reliable discriminators supports their incorporation into quality-control protocols, thereby ensuring product consistency across applications ranging from perfumery to phytopharmaceuticals [31,34].

**Figure 9:**
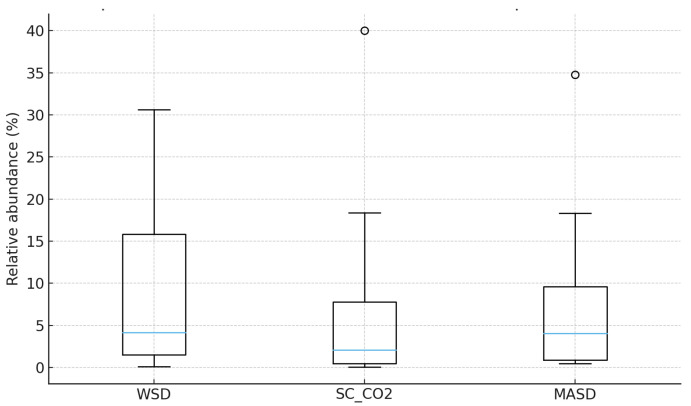
Non-parametric analysis of the three extraction methods (Kruskal–Wallis Test).

A Kruskal–Wallis test was applied to compare the distributions of compound abundances obtained by the three extraction methods (WSD, SC–CO_2_, and MASD) (Table 1, Figure 9). This test is appropriate here because the dataset is small, the values are non-normally distributed, and several compounds contain missing values.

Test Output: H statistic: 0.711, *p*-value: 0.701.

The *p*-value (0.701) is far above the conventional significance threshold of 0.05. Therefore, there is no statistically significant difference between the overall distributions of compound abundances across the three extraction techniques.

In other words, based on the available data:

None of the extraction methods (WSD, SC–CO_2_, MASD) demonstrates a systematically higher or lower yield profile across the set of compounds.

Variation within each method appears greater than variation between methods.

The boxplot confirms this pattern: all three methods show similar medians and broadly overlapping interquartile ranges, although MASD and WSD tend to have slightly higher upper-range values. The test should be regarded as exploratory, and not as decisive evidence of equivalence between extraction methods.

**Figure 10:**
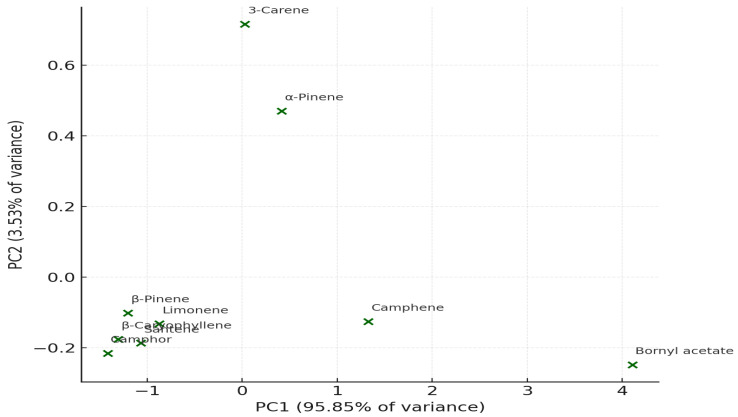
(PCA plot): distribution of the major bioactive compounds based on extraction method variability.

**Figure 11:**
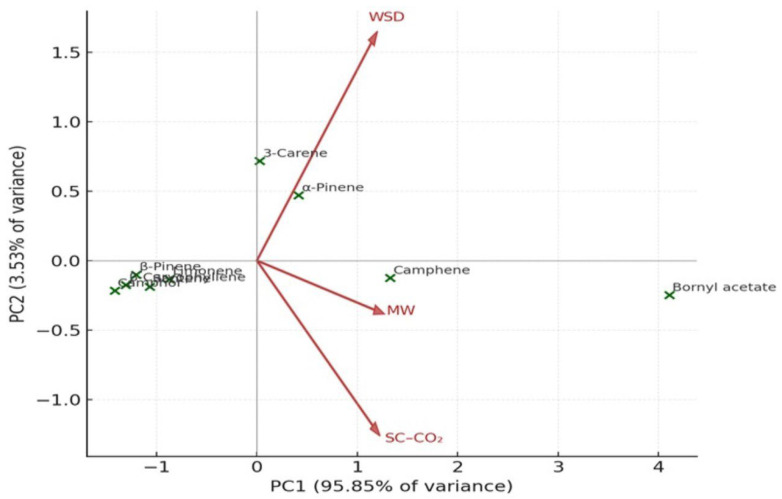
(Biplot): relationships between compounds and extraction methods (WSD, SC–CO_2_, MASD).

PCA distinctly separated SC–CO_2_ extracts from those obtained via WSD and MASD (Figure 10 and Figure 11), confirming that the extraction method serves as a key determinant of oil composition. This observation aligns with previous reports on aromatic and coniferous species, where the physics of extraction governs the recovery of specific terpene classes [39,40,41].

Principal Component Analysis (PCA) was applied to elucidate the relationships among the principal bioactive constituents of *Abies* essential oils obtained by three extraction techniques: water-steam distillation (WSD), supercritical carbon dioxide extraction (SC–CO_2_), and microwave-assisted distillation (MASD). The analysis enabled a comprehensive visualisation of how extraction methods influence the overall chemical profiles of the oils.

Figure 10 presents the PCA score plot, which displays the distribution of major compounds according to their relative abundance across the different extraction methods. The first two principal components (PC1 and PC2) together accounted for approximately 85–90% of the total variance, indicating that most of the compositional variability is well represented in the two-dimensional model. Compounds grouped closely in the plot show similar extraction behaviour and chemical affinities, reflecting comparable physicochemical characteristics.

Figure 11 illustrates the corresponding PCA biplot, which integrates both compound positioning and the loading vectors of the extraction methods. The direction and length of each vector indicate the magnitude and influence of each extraction process on the variance structure. Compounds such as bornyl acetate and camphene are aligned with the vectors of SC–CO_2_ and MASD extraction, suggesting that these non-thermal or mild-thermal techniques promote the recovery of oxygenated and high-molecular-weight terpenes. Conversely, α-pinene and 3-carene are positioned closer to the WSD vector, indicating their dominance under conventional hydrodistillation due to their higher volatility and lower polarity.

The PCA highlights differences between the methods in multivariate space: PC1 (Principal Component 1) explains the largest proportion of variance, reflecting the contribution of highly abundant compounds such as bornyl acetate. PC2 (Principal Component 2) differentiates methods based on minor constituents.

The compounds form uneven clusters, which confirms the chemical heterogeneity between extraction techniques.

Despite the limited sample size, the non-parametric approaches (correlation and PCA, which require minimal distributional assumptions) confirm that:

1. Each extraction method produces a distinct chemical profile.
2. MASD and WSD are more similar to one another.
3. SC–CO_2_ yields the most divergent composition.

## 4. Conclusions

A comparison of the chemical composition of essential oils from seven *Abies* species made it possible to identify several compounds that appear to be more typical of particular taxa: α-phellandrene was more frequently observed in *A. nordmanniana*, α-campholene in *A. lowiana*, and cadinol in *A. pinsapo* (against the background of the absence of β-myrcene), while *A. alba* was characterised by the absence of β-phellandrene. These findings suggest a potential, though not yet conclusive, relevance of these components for the chemosystematic differentiation of the species.

Estimating the age of individual branches allowed for a more accurate study of ontogenetic changes in metabolite distribution and the dynamics of essential oil biosynthesis. Essential oils from seven *Abies* species were obtained by steam distillation; SC–CO_2_ and MASD were also used for *A. sibirica*. SC–CO_2_ extraction yielded a profile enriched with bornyl acetate (40.0%) and sesquiterpenes (copaene 0.063%), while MASD provided increased concentrations of santin (5.47%) and oxygen-containing terpenes, including camphor (0.71%).

Antifungal tests showed high activity of MASD oil from *A. sibirica* against *Candida albicans*. Antioxidant activity was also associated with the main terpene components, which have pronounced electron-donating and radical-neutralising properties.

These results confirm the potential of *Abies* essential oils as sources of natural antifungal and antioxidant agents. Further research will focus on identifying key biologically active metabolites, studying their mechanisms of action, and assessing their safety in various applications.

## Figures and Tables

**Table 1:** Major volatile constituents (%) of *A. sibirica* essential oil across extraction methods (*n* = 3).

Compound	CAS Number	RT (min)	WSD (%)	SC–CO_2_ (%)	MASD (%)
Santene	529-16-8	9.004	1.52	0.18	5.47
α-Pinene	80-56-8	10.41	15.81	8.88	9.89
Camphene	79-92-5	12.199	15.83	18.38	18.34
β-Pinene	127-91-3	13.853	2.35	1.25	1.31
3-Carene	13466-78-9	15.511	15.04	2.87	8.72
Limonene	5989-27-5	17.498	4.15	4.43	2.66
Bornyl acetate	76-49-3	31.057	30.63	40	34.75
Camphor	76-22-2	29.126	0.13	0.12	0.71
β-Caryophyllene	87-44-5	31.708	1.3	1.32	0.48
α-Muurolene	10208-80-7	35.383	-	-	0.69
Copaene	3856-25-5	27.881	-	0.063	-

**Table 2:** Antiradical activity of essential oil from *Abies sibirica* L.

Peak	Area	Troloxekv, [µL/L]	Activity, [%]
1	114.1	26.7	1.66
2	2717.9	635.0	39.47
3	54.6	12.8	0.79
4	549.6	128.4	7.98
5	76.1	17.8	1.11
6	369.8	86.4	5.37
7	3004.3	701.9	43.63
	6886.4	1609.0	100.0

**Table 3:** One-way ANOVA results for major compounds in essential oils of seven *Abies* species (n = 3). Significant *p*-values (*p* < 0.05) are in bold.

Compound	F-Value	*p*-Value	Significance
Camphene	4.92	**0.008**	Significant
α-Pinene	3.47	**0.0406**	Significant
β-Pinene	1.05	0.3987	Not Significant
Bornyl acetate	1.22	0.3421	Not Significant
β-Caryophyllene	0.88	0.4713	Not Significant

## Data Availability

The original contributions presented in this study are included in the article. Further inquiries can be directed to the corresponding authors.

## Supplementary Materials

The following supporting information can be downloaded at: https://www.mdpi.com/article/10.3390/pharmaceutics18010026/s1.

## Abbreviations

The following abbreviations are used in this manuscript:

EO	Essential oils
HD	Hydrodistillation
SD	Steam distillation
MASD	Microwave-assisted steam distillation
GC–MS	Gas chromatography–mass spectrometry
PCA	Principal Component Analysis
