# Supplementary material for: Comparative Phytochemical Profiling of Essential Oils from Selected Abies Species and Analysis of Their Antifungal and Antiradical Activity
Source: Pharmaceutics. 2025 Dec 25;18(1):26. doi: 10.3390/pharmaceutics18010026 (PMC12845231; doi:10.3390/pharmaceutics18010026)
Supplement: Supplementary file 1 [file pharmaceutics-18-00026-s001.zip › Supplementary File S1.pdf]

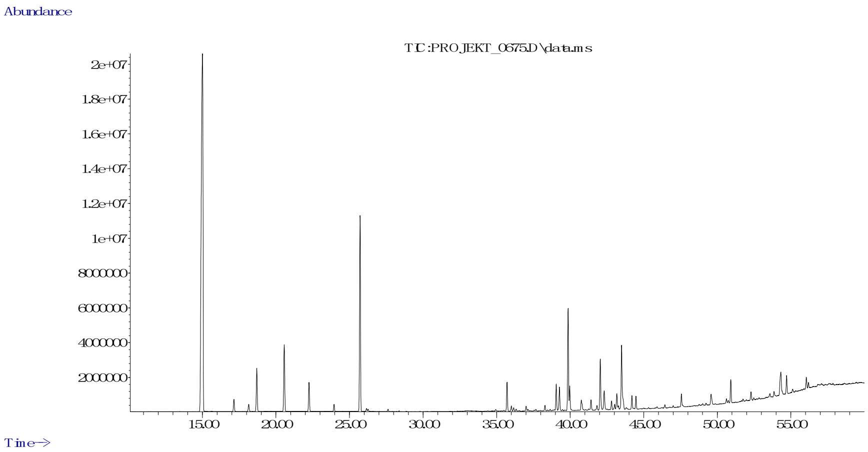

a) Abies alba

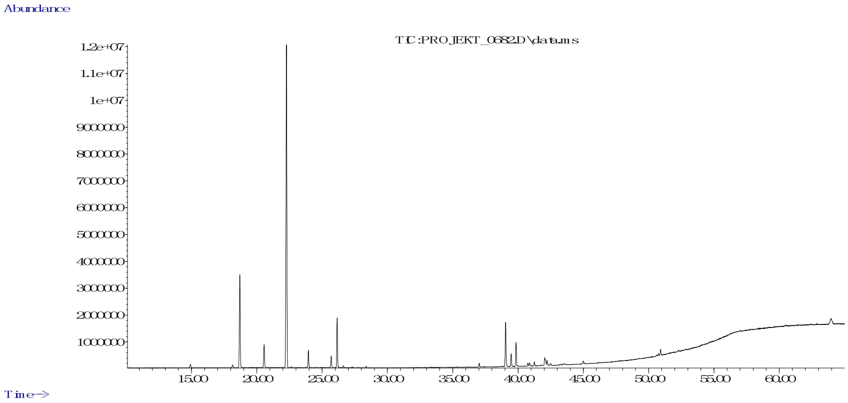

b) Abies concolor (1 year)

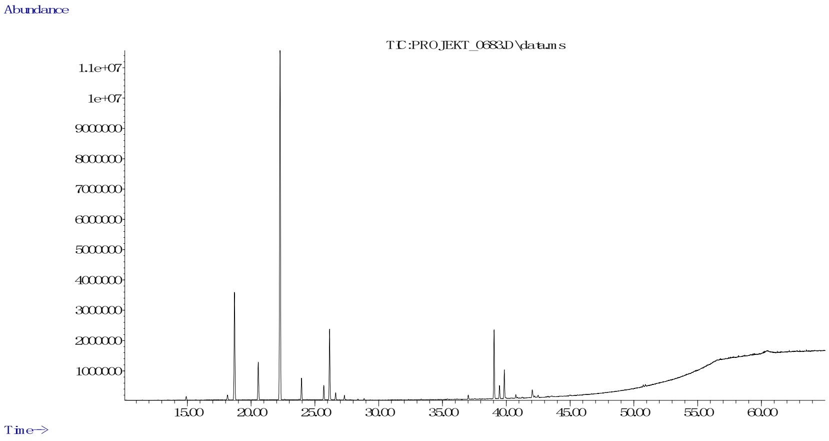

c) Abies concolor (3 year)

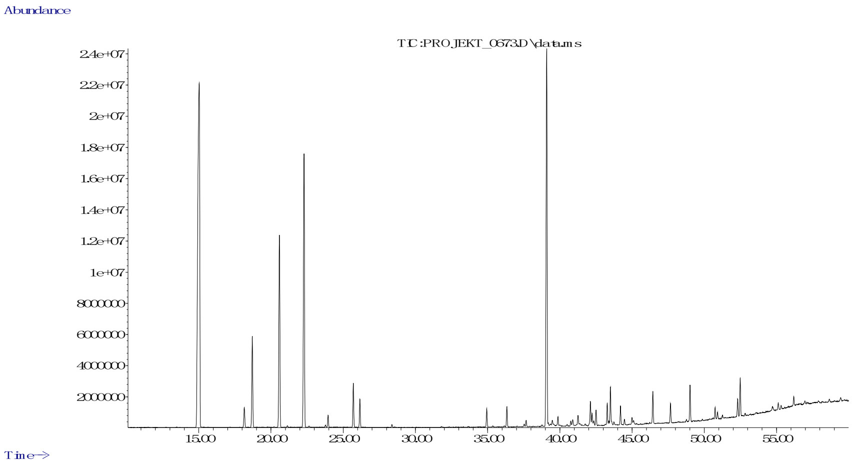

d) Abies grandis (1 year)

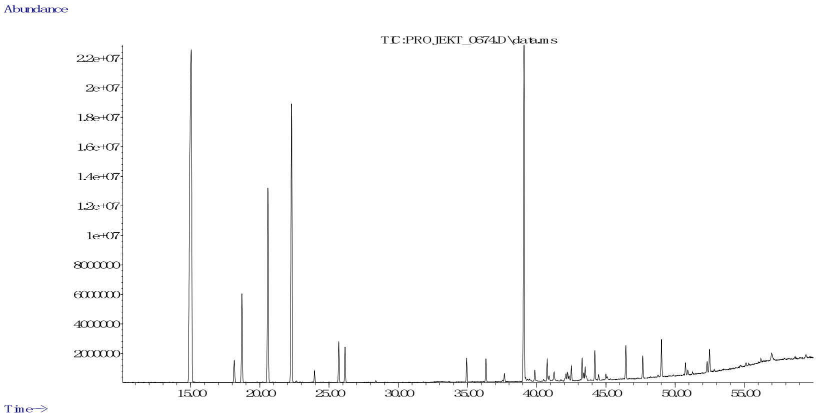

e) Abies grandis (3 year)

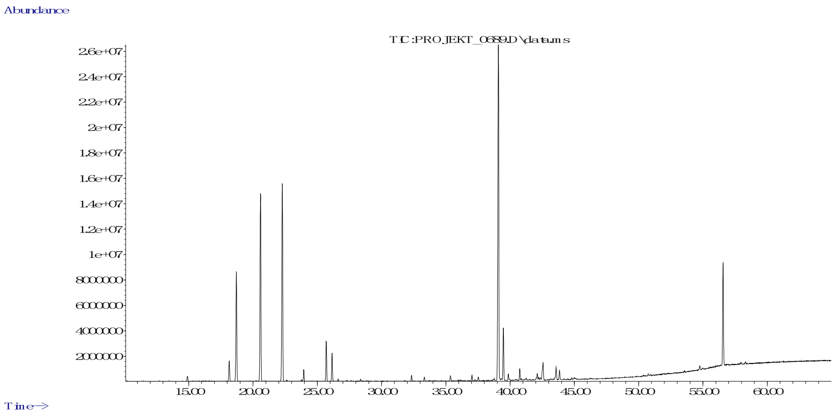

f) Abies lowiana (1 year)

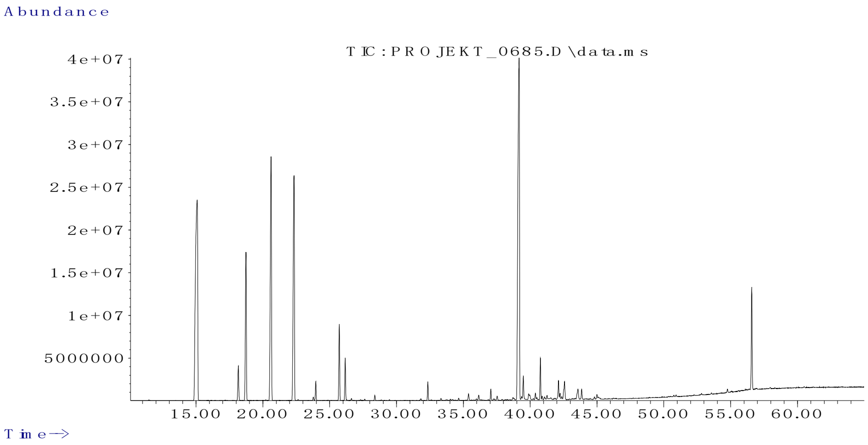

g) Abies lowiana (3 year)

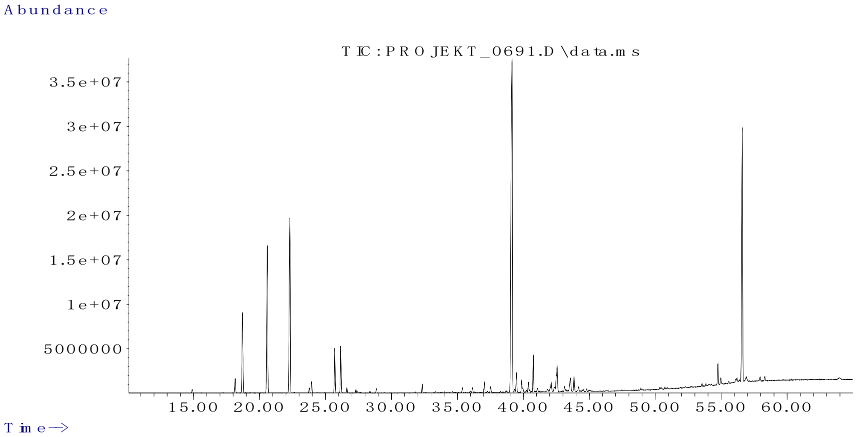

k) Abies lowiana (5 year)

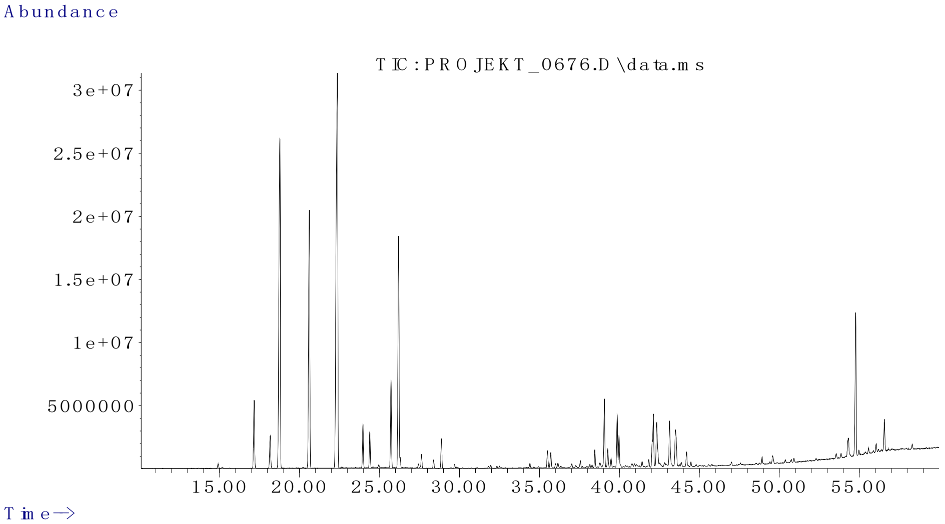

l) Abies nordmanniana (1 year)

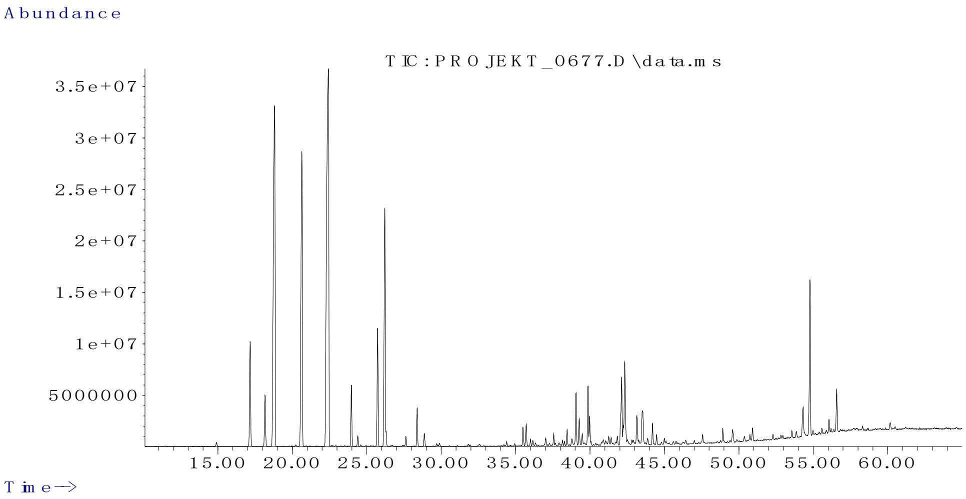

m) Abies nordmanniana (3 year)

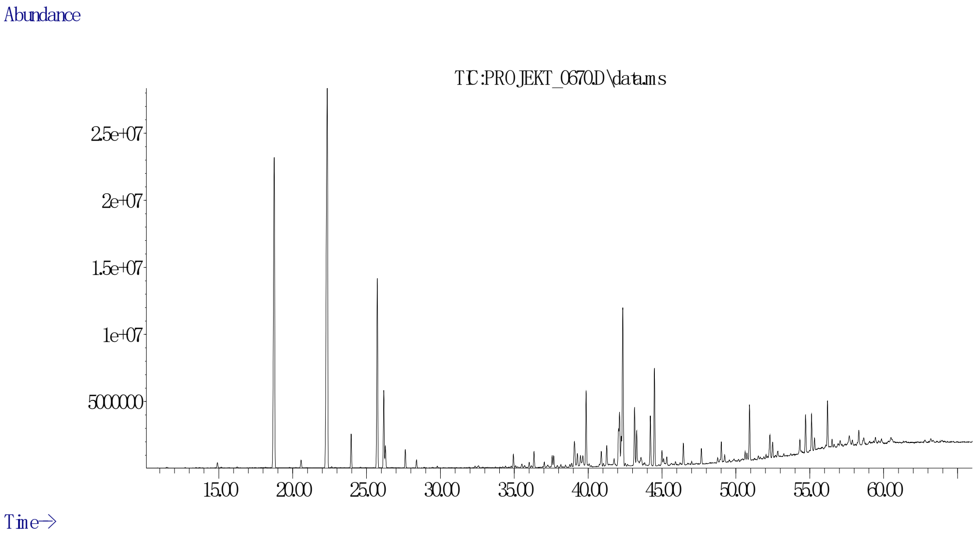

n) Abies pinsapo (1 year)

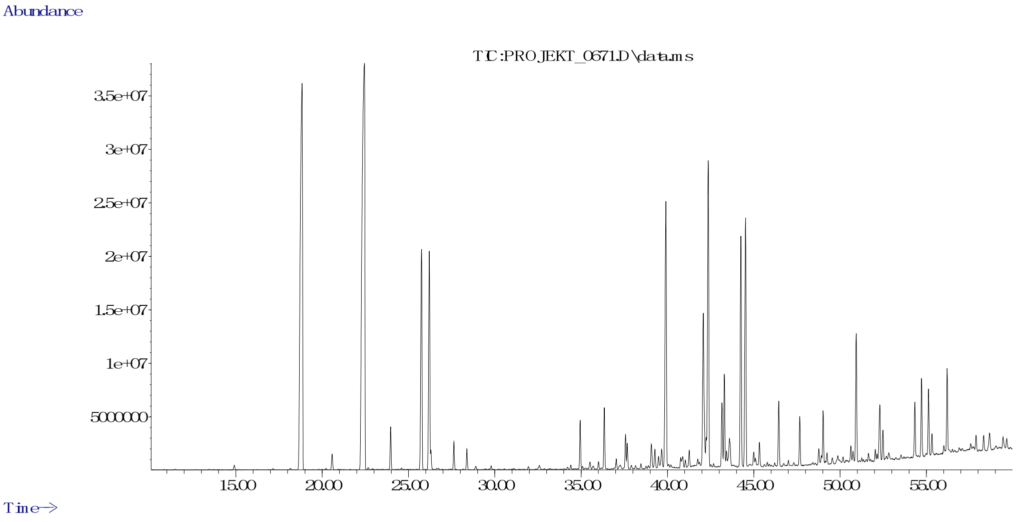

z) Abies pinsapo (3 year)

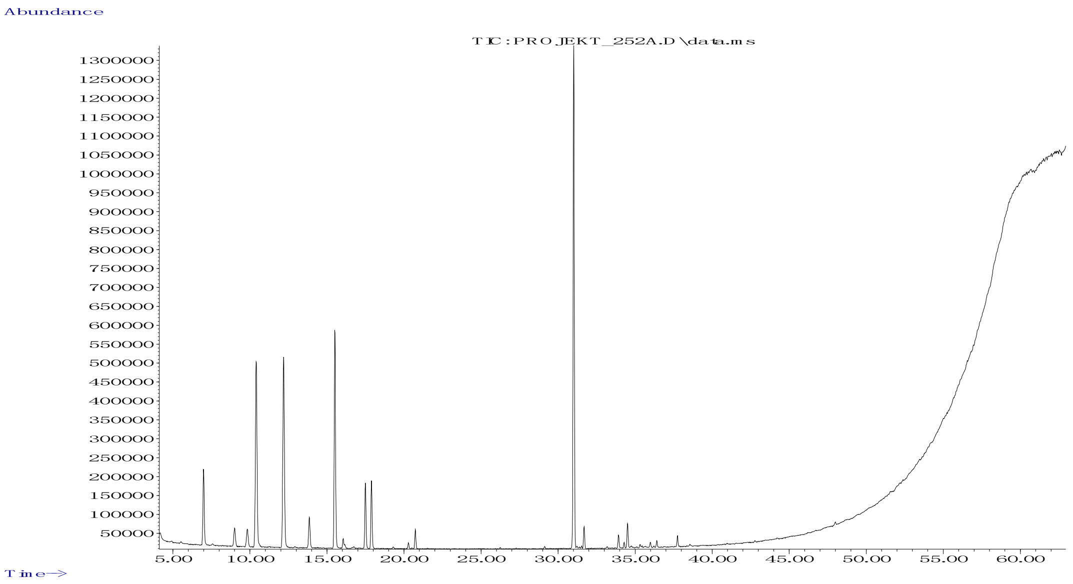

p) Abies sibirica

Figure S1. C-MS chromatogram of volatile components of essential oils extracted from different species *ABIES* method of water-steam distillation (a, b, c, d, e, f, g, k, l, m, n, z, p).

Table S1. Volatile components identified in *A. sibirica, A. pinsapo, A. grandis, A. concolor, A. nordmanniana, A. lowiana, A. alba.*

R.Time (min.)	Name	UPAC name	CAS Number	Molecular Weight	Code Number
17.158	Santene	2,3-dimethyl- Bicyclo[2.2.1]hept-2-ene	000529-16-8	122.11	1
18.162	Tricyclene	1,7,7-trimethyl- Tricyclo[2.2.1.0(2,6)]heptane	000508-32-7	136.13	2
18.765	α-Pinene	2,6,6-Trimethylbicyclo[3.1.1]hept-2-ene	007785-70-8	136.13	3
20.610	Camphene	2,2-dimethyl-3-methylene- Bicyclo[2.2.1]heptane	000079-92-5	136.13	4
22.362	β-Pinene	6,6-dimethyl-2-methylene-Bicyclo[3.1.1]heptane	000127-91-3	136.13	5
23.772		Unknown with Mr 136			6
23.960	3-Carene	3,7,7-trimethyl-Bicyclo[4.1.0]hept-3-ene	028634-89-1	136.13	7
23.963	β-Myrcene	7-methyl-3-methylene-1,6-Octadiene	000123-35-3	136.13	8
24.394	α-Phellandrene	2-methyl-5-(1-methylethyl)- 1,3-Cyclohexadiene	000099-83-2	136.13	9
25.720	Limonene	1-methyl-4-(1-methylethenyl)- Cyclohexene	000138-86-3	136.13	10
26.194	β-Phellandrene	3-methylene-6-(1-methylethyl)-Cyclohexene	000555-10-2	136.13	11
26.628	cis-Ocimene	3,7-dimethyl- (CAS)-1,3,7-Octatriene	006874-10-8	136.13	12
27.314	β-trans-Ocimene	3,7-dimethyl- (CAS)1,3,6-Octatriene	003779-61-1	136.13	13
27.430	ϒ-Terpinene	3-methylene-6-(1-methylethyl)- Cyclohexene	000555-10-2	136.13	14
27.627		Unknown with Mr 158			15
28.381	p-Cymene	1-methyl-3-(1-methylethyl)-Benzene,	000535-77-3	134.11	16
28.871	Terpinolene	1-methyl-4-(1-methylethylidene)-Cyclohexene	000586-62-9	136.13	17
31.822	α-Campholenal	2,2,3-trimethyl-3-Cyclopentene-1-acetaldehyde	004501-58-0	152.12	18
32.337	β-Nonanone	Methyl heptyl ketone	000821-55-6	142.14	19
33.331		1,3,3-trimethyl-Bicyclo[2.2.1]heptan-2-one	004695-62-9	152.12	20
34.406		Unknown with Mr 254			21
34.942	α- Cubebene		017699-14-8	204.19	22
35.499		Unknown with Mr 124			23
35.711		2,6,6,9-tetramethyl-Tricyclo[5.4.0.0(2,8)]undec-9-ene	005989-08-2	204.19	24
36.140		Unknown with Mr 204			25
36.334	α-Copaene	1,3-dimethyl-8-(1-methylethyl)-Tricyclo[4.4.0.0(2,7)]dec-3-ene	003856-25-5	204.19	26
37.023	Longicyclene	decahydro-1,5,5,8a-tetramethyl-1,2,4-Methenoazulene	001137-12-8	204.19	27
37.530	Camphor	1,7,7-trimethyl- (CAS)Bicyclo[2.2.1]heptan-2-one	000076-22-2	152.12	28

37.670	β- Cubebene	octahydro-7-methyl-3-methylene-4-(1-methylethyl)-1H-Cyclopenta[1,3]cyclopropa[1,2]benzene	013744-15-5	204.19	29
38.154		4-Isopropyl-1-methyl-2-cyclohexen-1-ol	029803-81-4	154.14	30
38.465		Unknown with Mr 122			31
38.785	Fenchol	1,3,3-trimethyl- Bicyclo[2.2.1]heptan-2-ol	001632-73-1	154.14	32
39.058	Bornyl acetate	1,7,7-trimethyl-Bicyclo[2.2.1]heptan-2-ol acetate	005655-61-8	196.15	33
39.133		Unknown with Mr 154			34
39.276		decahydro-4,8,8-trimethyl-9-methylene-1,4-Methanoazulene	000475-20-7	204.19	35
39.480	4-Terpineol	4-methyl-1-(1-methylethyl)-3-Cyclohexen-1-ol	000562-74-3	154.14	36
39.644		octahydro-7-methyl-3-methylene-4-(1-methylethyl)-1H-Cyclopenta[1,3]cyclopropa[1,2]benzene	013744-15-5	204.19	37
39.862	β-Caryophyllene	4,11,11-trimethyl-8-methylene-Bicyclo[7.2.0]undec-4-ene	000087-44-5	204.19	38
39.975	Himachala-2,4-diene	3,5,5,9-Tetramethyl-5,6,7,8,9,9a-hexahydro-1H-benzo[a]cycloheptene	060909-27-5	204.19	39
40.758		2,6-dimethyl-2,6-Octadiene	002792-39-4	138.14	40
40.804		7,11-dimethyl-3-methylene-1,6,10-Dodecatriene	028973-97-9	204.19	41
40.895		6,6-dimethyl-Bicyclo[3.1.1]hept-2-ene-2-carboxaldehyde	000564-94-3	150.10	42
40.942	Δ-Cadinene	1,2,3,5,6,8a-hexahydro-4,7-dimethyl-1-(1-methylethyl)- Naphthalene	000483-76-1	204.19	43
41.424		2,4a,5,6,7,8,9,9a-octahydro-3,5,5-trimethyl-9-methylene-1H-Benzocycloheptene	003853-83-6	204.19	44
41.838	α-Terpinene	1-methyl-4-(1-methylethyl)- 1,3-Cyclohexadiene	000099-86-5	136.13	45
42.049	α-Caryophyllene	2,6,6,9-tetramethyl- (CAS)-1,4,8-Cycloundecatriene	006753-98-6	204.19	46
42.121	α-Terpineol	alpha.,.alpha.,4-trimethyl-3-Cyclohexene-1-methanol	010482-56-1	154.14	47
42.238	Cryptone	4-(1-methylethyl)-2-Cyclohexen-1-one	000500-02-7	138.10	48
42.334	4-Carene	4,7,7-Trimethylbicyclo[4.1.0]hept-2-en	029050-33-7	136.13	49
42.351	α-Amorphene	1,2,4a,5,6,8a-hexahydro-4,7-dimethyl-1-(1-methylethyl)-Naphthalene	023515-88-0	204.19	50
42.511	Borneol	1,7,7-trimethyl-Bicyclo[2.2.1]heptan-2-ol	000507-70-0	154.14	51
42.565		2-Isopropenyl-4a,8-dimethyl-1,2,3,4,4a,5,6,7-octahydronaphthalene	000000-00-0	204.19	52
42.819		cis-(-)-2,4a,5,6,9a-Hexahydro-3,5,5,9-tetramethyl(1H)benzocycloheptene	000000-00-0	204.19	53
43.025	β-Bisabolene	1-methyl-4-(5-methyl-1-methylene-4-hexenyl)-(CAS)Cyclohexene	000495-61-4	204.19	54
43.138	Germacrene	1-methyl-5-methylene-8-(1-methylethyl)-1,6-Cyclodecadiene	023986-74-5	204.19	55
43.149	α-Cubebene	3a,3b,4,5,6,7-hexahydro-3,7-dimethyl-4-(1-methylethyl)1H-Cyclopenta[1,3]cyclopropa[1,2]benzene	017699-14-8	204.19	56
43.282	α-Muurolene	1-Isopropyl-4,7-dimethyl-1,2,4a,5,6,8a-hexahydronaphthalene	010208-80-7	204.19	57
43.397		1,2,3,4,4a,5,6,7-octahydronaphthalene8-Isopropyl-5-methyl-2-methylene	150320-52-8	204.19	58
43.506	Geranyl acetate	3,7-dimethyl-, acetate, 2,6-Octadien-1-ol	000105-87-3	196.15	59

43.581	β-Citronellol	3,7-dimethyl- (CAS)6-Octen-1-ol	000106-22-9	156.15	60
43.871	β-Panasinsene	2,2,4a-Trimethyl-8-methylenedecahydrocyclobuta[c]indene	000000-00-0	204.19	61
44.201	Δ-Cadinene	1,2,3,5,6,8a-hexahydro-4,7-dimethyl-1-(1-methylethyl)- (CAS)Naphthalene	000483-76-1	204.19	62
44.475	ϒ-Cadinene	1,2,3,4,4a,5,6,8a-octahydro-7-methyl-4-methylene-1-(1-methylethyl)-Naphthalene	039029-41-9	204.19	63
44.801		Unknown with Mr 204			64
44.998	Myrtenol	6,6-dimethyl-Bicyclo[3.1.1]hept-2-ene-2-methanol	000515-00-4	152.12	65
45.332	α-Cadinene		000000-00-0	204.19	66
46.439	Calamenene	1,2,3,4-tetrahydro-1,6-dimethyl-4-(1-methylethyl)-Naphthalene,	000483-77-2	202.17	67
46.665		Unknown with Mr 202			68
48.001	α-Bisabolol				69
48.925	1-Decene		000872-05-9	140.16	70
49.018	Δ-Cadinene	1,2,3,5,6,8a-hexahydro-4,7-dimethyl-1-(1-methylethyl)-Naphthalene	000483-76-1	204.19	71
49.584		1,2,3,6-Tetramethylbicyclo[2.2.2]oct-2-ene	062376-14-1	164.16	72
50.375		Unknown with Mr 152			73
50.747	Nerolidol	3,7,11-trimethyl- 1,6,10-Dodecatrien-3-ol	007212-44-4	222.20	74
50.918	Caryophyllene oxide	12-trimethyl-9-methylene-5-Oxatricyclo[8.2.0.0(4,6)]dodecane	001139-30-6	220.18	75
51.263		Unknown with Mr 222			76
52.312	α-Cubebene	3a,3b,4,5,6,7-hexahydro-3,7-dimethyl-4-(1-methylethyl)-1H-Cyclopenta[1,3]cyclopropa[1,2]benzene	017699-14-8	204.19	77
52.488		Unknown with Mr 204			78
52.717		Unknown with Mr 204			79
52.929		Unknown with Mr 222			80
53.560		1,2,3,4,4a,5,6,7-octahydro-.alpha.,.alpha.,4a,8-tetramethyl-2-Naphthalenemethanol	001209-71-8	222.20	81
53.836		Unknown with Mr 222			82
53.864	α-Cedren		035944-22-0	204.19	83
54.318		Unknown with Mr 222			84
54.719		2-isopropyl-5-methyl-9-methylene-Bicyclo[4.4.0]dec-1-ene	150320-52-8	204.19	85
54.73	α-Cadinol	4-Isopropyl-1,6-dimethyl-1,2,3,4,4a,7,8,8a-octahydro-1-naphthalenol	000481-34-5	222.20	86
54.754	Selina-6-en-4-ol	7-Isopropyl-1,4a-dimethyl-1,2,3,4,4a,5,6,8a-octahydro-1-naphthalenol	000000-00-0	156.15	87
54.853		Unknown with Mr 156			88
55.121	α-Copaene	1,3-dimethyl-8-(1-methylethyl)-Tricyclo[4.4.0.0(2,7)]dec-3-ene	003856-25-5	204.19	89
55.125	Cadinol	4-Isopropyl-1,6-dimethyl-1,2,3,4,4a,7,8,8a-octahydro-1-naphthalenol	000481-34-5	222.20	90
55.322	α-Cubebene	3a,3b,4,5,6,7-hexahydro-3,7-dimethyl-4-(1-methylethyl)-1H-Cyclopenta[1,3]cyclopropa[1,2]benzene	017699-14-8	204.19	91
55.414		Unknown with Mr 204			92

55.644		Unknown with Mr 204			93
55.982		Unknown with Mr 204			94
56.058		Unknown with Mr 204			95
56.522		Unknown with Mr 204			96
56.569	Δ-Selinene	2,3,4,4a,5,6-hexahydro-1,4a-dimethyl-7-(1-methylethyl)- (CAS)Naphthalene	028624-28-4	204.19	97
56.901		Unknown with Mr 204			98
57.954		Unknown with Mr 204			99
59.444		Unknown with Mr 204			100
60.524		Unknown with Mr 204			101

continuation of table S1

**Code Number**	**Abies concolor (1year),%**	**Abies concolor (3 year),%**	**Abies grandis (1 year),%**	**Abies grandis (3 year),%**	**Abies lowiana (1 year),%**	**Abies lowiana (3 year),%**	**Abies lowiana (5 year),%**	**Abies normanniana (1 year),%**	**Abies normanniana (3 year),%**	**Abies pinsapo (1 year),%**	**Abies pinsapo (3 year),%**	**Abies sibirica,%**	**Abies alba,%**
1								2.44	2.98			1.52	0.72
2		0.67	1.09	1.01	1.58	1.87	0.84	1.21	1.51			1.6	0.45
**3**	**14**	**14.09**	**4.91**	**4.05**	**8.85**	**6.82**	**4.82**	**16.27**	**16.12**	**15.04**	**14.4**	**15.81**	**2,58**
**4**	**3.47**	**4.94**	**11.21**	**9.45**	**16.3**	**12.53**	**9.72**	**11.04**	**11.35**	**0.29**	**0.28**	**15.83**	**4,08**
5	51.28	48.39	16.95	14.83	17.4	12.67	12.3	23.72	22.86	21.47	18.6	2.35	1,71
6			0.1	0.43			0.26						
7										1.08	0.67	15.04	0.36
8	2.25	2.4	0.55	0.43	0.76	0.86	0.55	1.33	1.45			0.82	
9								1.14	0.25				
10	1.59	1.68	2.13	1.63	2.73	3.24	2.36	2.93	3.12	7.16	4.62	4.15	11,33
11	6.67	8.38	1.35	1.42	1.94	1.94	2.51	9.11	7.78	2.68	4.58	4.32	
12		0.78				0.11	0.24			0.71			
13		0.48					0.15						
14								0.15				0.1	
15								0.45	0.24	0.58	0.43		
16						0.26		0.26	0.91	0.27	0.33	0.35	

17							0.22	0.94	0.34			1.11	
18						0.12					0.05		
19					0.37	0.81	0.4						
20					0.28	0.13	0.27						
21								0.16	0.12		0.07		
22			0.86	0.93						0.41	0.78		
23					0.41	0.43		0.6	0.52	0.14	0.16		
24								0.59	0.59		0.08		1,58
25							0.26	0.13	0.16	0.18	0.13		
26			0.96	0.91		0.32			0.09	0.54	1.02		
27					0.35	0.53	0.48	0.17	0.25	0.18	0.19		0.25
28					0.28	0.27	0.36	0.23	0.3	0.4	0.58	0.13	
29			0.31	0.33						0.38	0.4		
30								0.11	0.13		0.06		0.29
31								0.53	0.37		0.08		
32					0.12	0.26		0.23	0.27	0.13	0.05		
**33**	**6.45**	**8.02**	**0.67**	**16.25**	**30.94**	**21.19**	**32.72**	**2.31**	**1.44**	**1.16**	**0.51**	**30.63**	**1,53**
34			0.4	0.42									
35								0.61	0.72	0.5	0.37		1,37
36	1.86	1.55			3.86	0.7	1.07	0.28	0.34	0.42	0.23	0.12	
37										0.49	0.43		
38	3.26	3.19	0.45	0.42	0.48	0.26	0.85	1.69	1.5	2.59	5.92	1.3	5.83
39					0.09	0.76	0.52	1.07	0.78				1.29
40			0.4	0.82	0.7	1.91	1.81				0.16		0.15
41								0.14	0.29				
42			0.57	0.25						0.65	0.28		
43								0.08		0.1			
44			0.66	0.22				0.16	0.31	0.81	0.36		0.53
45							0.35		0.34	0.58	0.12		
46	1.69	1.09									3.68	0.81	2,99

47			2.12	0.38	0.6	1.59	0.82	2.41	2.3	3.06		0.3	
48			0.38	0.39									
49									3.15		6.77		1.26
50				0.22			0.46	2.16		7.25			
51			0.74	0.59				0.2	0.14			1.44	
52					1.82	1.41	2.19						
53								0.07	0.2				0.48
54												0.2	0.30
55							0.3	1.78		2.09	1.18		0.96
56									1.11				
57			0.91	0.88						1.38	1.56		0.18
58			0.25	0.26							0.29	0.1	
59			1.84	0.7			1.23	1.94	1.53		0.84	0.32	4.57
60					1.07	1.07				0.51			
61			0.13	0.44	0.7	0.59	0.87		0.21				
62			0.85	0.85			0.25	0.53	0.55	1.68	4.4		0.75
63			0.25	0.49				0.16	0.25	3.32	4.9		0.64
64							0.15		0.07				
65			0.59	0.16		0.29			0.15	0.55	0.3		
66										0.31	0.44		
67			1.51	1.39					0.13	0.75	1.08		0.19
68			0.91	0.91					0.3		0.06		0.86
69												0.27	
70								0.24	0.36	0.21			
71			1.71	1.49						0.81	1.02		
72								0.33	0.43	0.31	0.13		0.8
73									0.18	0.34	0.06		0.28
74			0.53	0.45				0.11	0.23	0.27	0.22		0.16
75	0.85		0.3	0.2		0.11		0.14	0.35	1.95	2.39		1,31
76			0.16	0.05						0.14	0.07		

77			0.83	0.43					0.15	0.96	1.15		0.51
78			1.81	0.89						0.53	0.49		0.13
79		0,65				0.14			0.11	0.25	0.08		0.18
80		0.12							0.08		0.15		
81						0.07	0.15	0.18	0.2		0.07		0.35
82		0.3											0.36
83								0.17	0.17				
84									1.25	0.78	0.97		2,27
85			0.42	0.05							1.29		1,05
86										1.35			
87		0.23			0.33	0.21	1.13	5.11	4.43				
88		0.04							0.15		0.07		
89			0.38	0.15					0.06		1.11		
90										1.51	0.28		0.25
91			0.19	0.43						0.56	0.41		
92		0.21							0.09				
93		0.19							0.15				
94		0.32							0.1	0.74			0.34
95			0.44	0.15				0.32	0.37	2.38	1.62	0.74	0.74
96		0.9								0.41	0.1		
97					7.63	4.25	16.62	1.05	1.14	0.32	0.11		
98							0.56		0.03	0.35	0.19		0.72
99							0.27	0.18	0.07	0.31	0.31		0.28
100			0.24	0.31						0.99	0.28		
101		1.39											
