# Supplementary material for: Comparative Phytochemical Profiling of Essential Oils from Selected Abies Species and Analysis of Their Antifungal and Antiradical Activity
Source: Pharmaceutics. 2025 Dec 25;18(1):26. doi: 10.3390/pharmaceutics18010026 (PMC12845231; doi:10.3390/pharmaceutics18010026)
Supplement: Supplementary file 1 [file pharmaceutics-18-00026-s001.zip › Supplementary File S2.pdf]

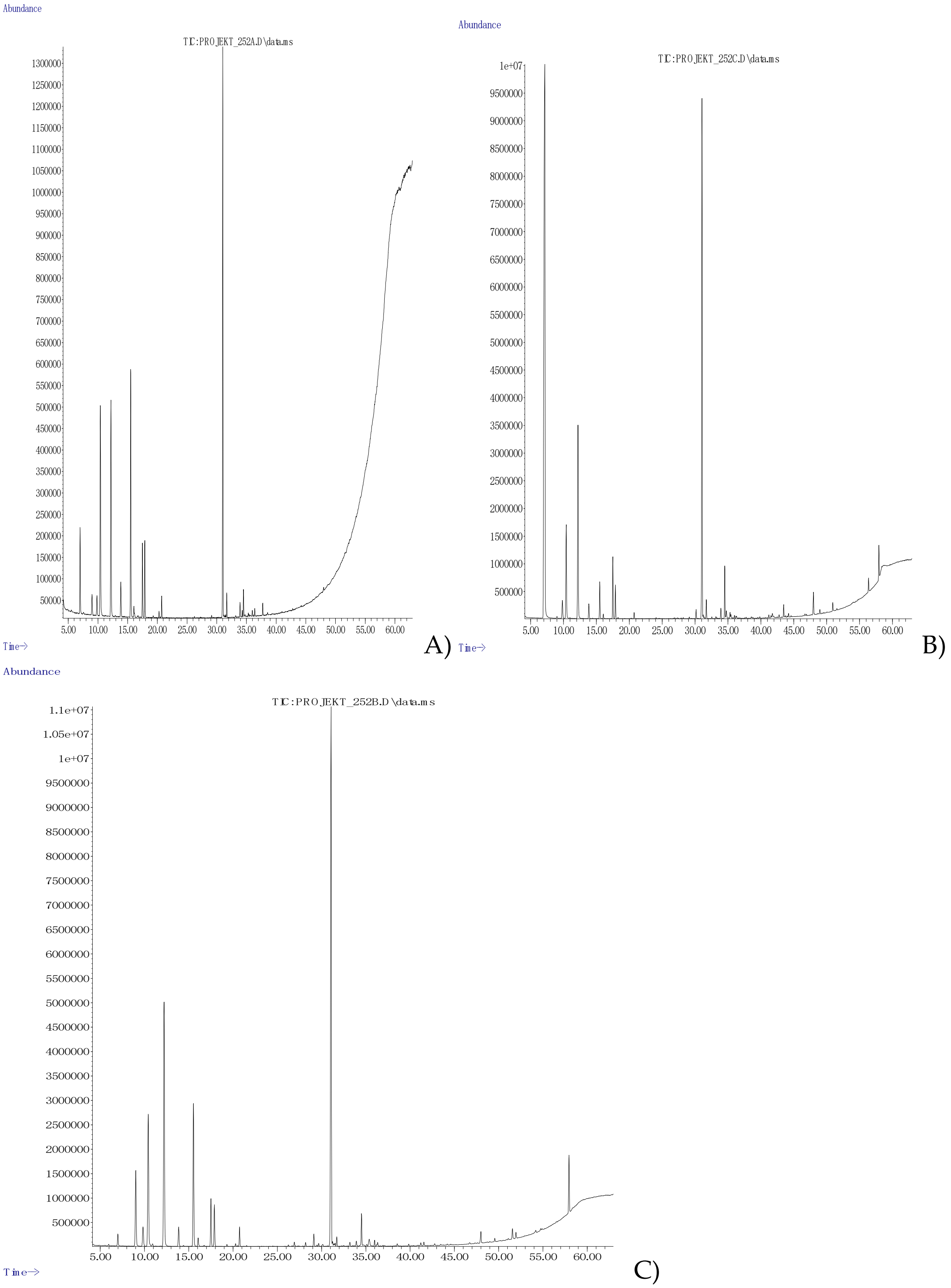

Figure S2 GC-MS chromatogram of volatile components of essential oils extracted from *Abies sibirica L.* method of water-steam distillation (A), the carbon-dioxide extraction (B), the microwave-assisted steam distilation (C).

Table S2. Volatile components identified in *Abies sibirica.*

Name	CAS Number	IUPAC name	RT, min	Relativ % Water Steam distillation	Relativ Con,%, Carbon dioxide exraction	Relativ.con. %, Microwave exraction
Santene	529-16-8	2,3-dimethyl-Bicyclo[2.2.1]hept-2-ene	9.004	1.517	0,183	5,470
Tricyclene	508-32-7	1,7,7-trimethyl-Tricyclo[2.2.1.0(2,6)]heptane	9.829	1.603	1,819	1,515
α-Pinene	80-56-8	2,6,6-trimethyl-Bicyclo[3.1.1]hept-3-ene	10.410	15.807	8,875	9,891
1-propanol			10.977		0,062	0,160
Camphene	79-92-5	2,2-dimethyl-3-methylene-Bicyclo[2.2.1]heptane	12.199	15.831	18,384	18,336
β-Pinene	127-91-3	6,6-dimethyl-2-methylene-Bicyclo[3.1.1]heptane	13.853	2.345	1,254	1,310
3-Carene	13466-78-9	3,7,7-trimethyl-Bicyclo[4.1.0]hept-3-ene	15.511	15.037	2,874	8,723
β-Myrcene	123-35-3	7-methyl-3-methylene-1,6-octadiene	16.051	0.819	0,364	0,512
Limonene	5989-27-5	1-methyl-4-prop-1-en-2-yl-cyklohexen	17.498	4.153	4,433	2,657
β-Phellandrene	555-10-2	3-methylene-6-(1-methylethyl)cyclohexene	17.889	4.319	2,412	2,330
2-hexenal	6728-26-3		18.287		0,091	
γ-Terpinene	99-85-4	1-methyl-4-(methylethyl)-1,4-Cyclohexadiene	19.298	0.101		0,107
p-Cymene	99-87-6	1-methyl-4-(1-methylethyl)-Benzene	20.284	0.354	0,053	0,151
4-Carene	586-62-9 or 29050-33-7	4,7,7-trimethyl-Bicyclo[4.1.0]hept-2-ene	20.512			0,036
Terpinolene	586-62-9	1-methyl-4-(1-methylethylidene)-Cyclohexene	20.733	1.111	0,414	1,011
Unknown with Mr 138			21.531			0,047
3-hexen-1-ol	928-96-1		24.072		0,083	
p-Cymenene	1195-32-0	1-methyl-4-(1-methylethenyl)-Benzene	26.252			0,080
Acetic acid	64-19-7		26.774		0,057	
α-Cubebene	17699-14-8	4,10-dimethyl-7-isopropyltricyclo[4.4.0.01,5]deca-3-ene	26.916		0,073	0,241
I-Menthone	14073-97-3	trans-2-isopropyl-5-methylcyclohexanone	27.285		0,077	
α-Longipinene	5989-08-2	2,6,6,9-tetramethyl-Tricyclo[5.4.0.0(2,8)]undec-9-ene	27.445		0,031	
Copaene	3856-25-5	1,3-dimethyl-8-(1-methylethyl)-Tricyclo[4.4.0.0.02,7]dec-3-ene	27.881		0,063	
α-Copaene	3856-25-5	1,3-dimethyl-8-(1-methylethyl)-Tricyclo[4.4.0.0.02,7]dec-3-ene	28.203		0,079	0,210
Camphor	76-22-2	1,7,7-trimethyl-Bicyclo[2.2.1]heptan-2-one	29.126	0.133	0,121	0,712
Unknown with Mr 204			29.480			0,079
β-Cubebene	013744-15-5	octahydro-7-methyl-3-methylene-4-(1-methylethyl)-1Hcyclopenta(1,3)cyclopropa(1,2)benzene	29.670			0,168
Unknown with Mr 204			30.154		0,672	0,138
Bornyl acetate	76-49-3	1,7,7-trimethyl-Bicyclo[2.2.1]heptan-2-yl acetate	31.057	30.627	40,000	34,754
Unknown with Mr 164			31.231	0.150	0,263	0,250
Unknown with Mr 170			31.364		0,139	0,164
Terpinen-4-ol	562-74-3	4-methyl-1-(1-methylethyl)-3-Cyclohexen-1-ol	31.527	0.119	0,058	0,154
β-Caryophyllene	87-44-5	4,11,11-trimethyl-8-methylene-bicyclo[7.2.0)]undec-4-ene	31.708	1.298	1,315	0,484
Unknown with Mr 134			32.542		0,154	0,056

Unknown with Mr 136			33.198	0.139		0,238
Unknown with Mr 136			33.202		0,139	
Unknown with Mr 204			33.322		0,050	
Unknown with Mr 204						0,043
α-Caryophyllene	6753-44-5	2,6,6,9-tetramethyl-1,4,8-Cycloundecatriene	33.923	0.805	0,734	0,293
α-Terpineol	98-55-5	2-(4-methyl-1-cyclohex-3-enyl)propan-2-ol	34.286	0.295		
Borneol	507-70-0	1,7,7-trimethyl- Bicyclo[2.2.1]heptan-2-ol	34.510	1.435	3,462	1,759
Unknown with Mr 204			34.756			0,118
Unknown with Mr 2044			35.111		0,765	
β-Bisabolene	495-61-4	1-Methyl-4-(6-methylhepta-1,5-dien-2-yl)cyclohex-1-ene	35.318	0.202	0,409	
α-Muurolene	10208-80-7	1-isopropyl-4,7-dimethyl-1,2,4a,5,6,8a-hexahydronaphthalene	35.383			0,686
Unknown with Mr 204			35.451	0.102	0,282	
D-Carvone	2244-16-8	(*S*)-5-Isopropenyl-2-methyl-2-cyclohexenone	35.737		0,061	
Geranyl acetate	105-87-3	3,7-dimethyl-2,6-Octadien-1-ol, acetate	35.989	0.318	0,197	0,293
Unknown with Mr 204			36.222	0.076	0,173	0,055
δ-Cadinene	483-76-1	1-isopropyl-4,7-dimethyl-1,2,3,4,5,6,8a-hexahydronaphthalene	36.332		0,123	0,194
2,4-Dodecadienal	25152-84-5		36.406	0.415		
Unknown with Mr 204			36.643		0,045	
Unknown with Mr 204			36.829		0,050	
Methyl salicilate	119-36-8	Methyl 2-hydroxybenzoate	36.937			0,046
2-Tridecanone	593-08-8		37.555		0,039	
Unknown with Mr 204			37.768	0.618	0,082	
Calamenene	483-77-2	4-isopropyl-1,6-dimethyl-1,2,3,4,-tetrahydronaphthalene	38.544		0,086	0,108
Unknown			38.646		0,076	
Unknown			38.824		0,053	
Unknown			39.544		0,066	
Unknown			39.840		0,090	0,098
Unknown with Mr 222			41.212		0,209	0,163
Unknown with Mr 140			41.550		0,118	0,199
Maltol	118-71-8	3-Hydroxy-2-methyl-4H-pyran-4-one	41.754		0,462	
Unknown with Mr 140			41.983		0,114	
Caryophyllene oxide	1139-30-6	4,12,12-trimethyl-9-methylene-5-oxatricyclo[8.2.0.04,6]-dodecane	42.774		0,195	0,102
Nerolidol	7214-44-4	3,7,11-trimethyl-1,6,10-dodecatrien-3-ol	43.470		0,777	0,046
Unknown with Mr 204			43.761		0,092	
Unknown			44.022		0,086	0,102
Unknown with Mr 204			44.214		0,272	0,062
Unknown with Mr 204			44.594		0,088	
Unknown with Mr 204			46.697		0,152	0,094
Unknown with Mr 204			46.939		0,085	

α-Bisabolol	515-69-5	6-Methyl-2-(4-methyl-3-cyclohexen-1-yl)-5-hepten-2-ol	47.999	0.270	1,837	0,745
Farnesol acetate	29548-30-9	3,7,11-trimethy-2,6,10-dodecatrien-1-ol acetate	48.984		0,310	
Unknown with Mr 272			49.565			0,189
Farnesol	4602-84-0	3,7,11-trimethy-2,6,10-dodecatrien-1-ol	50.934		0,598	
Epimanoyl oxide	596-84-9	3-ethenyldodecahydro-3,4a,7,7,10a-pentamethyl-1H-Naphtho[2,1-b]pyran	51.560			0,587
Epimanoyl oxide (isomer)	27642-41-7	3-ethenyldodecahydro-3,4a,7,7,10a-pentamethyl-1H-Naphtho[2,1-b]pyran	51.955			0,356
Phytol	150-86-7	3,7,11,15-Tetramethyl-2-hexadecen-1-ol	56.370		0,926	
Manool	596-85-0	.alpha.-ethenyldecahydro-.alpha.,5,5,8a-tetramethyl-2-methylene-1-Naphthalenepropanol	57.947		2,225	3,001
